# High-Density Genetic Linkage Maps Provide Novel Insights Into ZW/ZZ Sex Determination System and Growth Performance in Mud Crab (*Scylla paramamosain*)

**DOI:** 10.3389/fgene.2019.00298

**Published:** 2019-04-05

**Authors:** Khor Waiho, Xi Shi, Hanafiah Fazhan, Shengkang Li, Yueling Zhang, Huaiping Zheng, Wenhua Liu, Shaobin Fang, Mhd Ikhwanuddin, Hongyu Ma

**Affiliations:** ^1^Guangdong Provincial Key Laboratory of Marine Biotechnology, Shantou University, Shantou, China; ^2^STU-UMT Joint Shellfish Research Laboratory, Shantou University, Shantou, China; ^3^Laboratory for Marine Fisheries Science and Food Production Processes, Pilot National Laboratory for Marine Science and Technology (Qingdao), Qingdao, China; ^4^Institute of Tropical Aquaculture, Universiti Malaysia Terengganu, Kuala Terengganu, Malaysia

**Keywords:** genetic linkage map, sex-specific SNP, QTL, association analysis, sex determination system, *Scylla paramamosain*

## Abstract

Mud crab, *Scylla paramamosain* is one of the most important crustacean species in global aquaculture. To determine the genetic basis of sex and growth-related traits in *S. paramamosain*, a high-density genetic linkage map with 16,701 single nucleotide polymorphisms (SNPs) was constructed using SLAF-seq and a full-sib family. The consensus map has 49 linkage groups, spanning 5,996.66 cM with an average marker-interval of 0.81 cM. A total of 516 SNP markers, including 8 female-specific SNPs segregated in two quantitative trait loci (QTLs) for phenotypic sex were located on LG32. The presence of female-specific SNP markers only on female linkage map, their segregation patterns and lower female: male recombination rate strongly suggest the conformation of a ZW/ZZ sex determination system in *S. paramamosain*. The QTLs of most (90%) growth-related traits were found within a small interval (25.18–33.74 cM) on LG46, highlighting the potential involvement of LG46 in growth. Four markers on LG46 were significantly associated with 10–16 growth-related traits. BW was only associated with marker 3846. Based on the annotation of transcriptome data, 11 and 2 candidate genes were identified within the QTL regions of sex and growth-related traits, respectively. The newly constructed high-density genetic linkage map with sex-specific SNPs, and the identified QTLs of sex- and growth-related traits serve as a valuable genetic resource and solid foundation for marker-assisted selection and genetic improvement of crustaceans.

## Introduction

Mud crab, *Scylla paramamosain* (Crustacea: Decapoda: Brachyura) is naturally distributed in the coasts of Asia regions along with other *Scylla* species (Waiho et al., 2016a, 2018; Fazhan et al., 2017a) and is the dominating mud crab species in the Mekong Delta, Vietnam (Le Vay et al., 2001), the southern part of Japan (Ogawa et al., 2012) and China (Ma et al., 2012). It is one of the main aquaculture invertebrate species in China, with a total production of more than 145,000 tons in 2016 (Department of Agriculture of China, 2017). Their high market values are attributed to their delicate meat, high nutritional value, ease of capture and hardy nature (Waiho et al., 2017). Thus, the development of genetic breeding programs, including marker- and gene-assisted selection are urgently needed to ensure sustainability and genetic variability of cultured mud crab. Attempts have been made to identify the correlation between some economically important growth traits of *S. paramamosain* such as body size and weight (Jiang et al., 2014), and transcriptome-derived microsatellite markers (Ma et al., 2014). These serve as foundations for further selection of genomic loci or genes related to the traits of interests by the construction of genetic maps and are of utmost importance and relevance to the aquaculture of this species.

Mud crabs exhibit significant sexual dimorphism, with females displaying higher growth rate and greater body weight compared to their male counterparts of the same size (Jiang et al., 2014; Waiho et al., 2016b). In addition, coupled with the gravid status of the mature females, the commercial value is substantially higher in females than in males. Therefore, it is of great interest to consider the possibility of mud crab monosex culture in near future. The first step of monosex culture is to understand its sex determination system. Unlike vertebrates, crustaceans’ sex determination systems are more diverse and plastic, influenced by both genetic and environmental factors (Ford, 2008). In crabs, both XX–XY and ZZ–ZW sex determination systems have been reported and is species-specific (Niiyama, 1938; Lécher et al., 1995; Cui et al., 2015). Based on our previous study using single-nucleotide polymorphisms (SNPs), that of *S. paramamosain* is postulated to be ZZ-ZW (Shi et al., 2018). Based on their transcriptomic profiles, some sex-related genes such as *vasa*, *Dmrt*, *FEM1*, and *Wnt6* were found to be differentially expressed in the gonad of *S. paramamosain* (Gao et al., 2014). Recently, we have also uncovered 147 gonadal differentially expressed long non-coding RNAs (lnc RNAs), nine of which showed regulation toward eight sex-related genes in *S. paramamosain* (Yang et al., 2017). The genome organization of sex chromosomes in *S. paramamosain* and the exact gene localization, however, remains unclear. The screening of sex-associated SNP markers will hasten the development of all-female *S. paramamosain* culture and contribute significantly to the understanding of the mud crab sex determination mechanism.

An accurate and comprehensive genetic linkage map is the cornerstone for genomic and genetic studies, as well as genetic breeding of a species. It aids in the elucidation of genomic characteristics, provides excellent framework for quantitative trait locus (QTL) localization, facilitates both marker- and gene-assisted selection, and enables comparative genome analysis between species (Yu et al., 2015; Peng et al., 2016). For example, sex-determination and/or growth-related traits were successfully mapped and studied in several aquaculture fish species, including the common carp (Peng et al., 2016), turbot (Taboada et al., 2014; Wang W. et al., 2015), blunt snout bream (Wan et al., 2017), bighead carp (Fu et al., 2016), Asian seabass (Wang L. et al., 2015), mandarin fish (Sun et al., 2017) and tilapia (Liu et al., 2013; Palaiokostas et al., 2015a). In decapod crustaceans, however, progress on the construction of high-density linkage maps is slow and difficult due to their high number of chromosomes. To date, SNP-based high-density linkage maps with thousands of markers and average marker distances of less than 1 cM are reported for only four decapod species (Baranski et al., 2014; Yu et al., 2015), of which two are portunid crabs – the Chinese mitten crab, *Eriocheir sinensis* (Cui et al., 2015; Qiu et al., 2017) and the swimming crab, *Portunus trituberculatus* (Lv et al., 2017).

Recently, with the advent of next-generation sequencing (NGS) technologies, SNPs may be mined using specific-locus amplified fragment sequencing (SLAF-seq) method, a modification of the commonly used restriction site-associated DNA sequencing (RAD-seq) method (Sun et al., 2013). This method involves size selection of restriction fragments to ensure even distribution and exclude repeats. SLAF-seq is gaining attention and has been used in plants (Xu et al., 2015; Luo et al., 2016; Zhou et al., 2017) and animals (Wang W.H. et al., 2015; Lv et al., 2017; Qiu et al., 2017) alike, especially those without reference genome, to construct high-density genetic maps as it is more efficient and cost-effective compared to the previous RAD-seq method (Qiu et al., 2017). The first genetic linkage map for *S. paramamosain* was constructed using microsatellite and amplified fragment length polymorphism (AFLP) markers (Ma et al., 2016). The resolution of the resulting map, however, is low (only 50% coverage of the estimated genome), with a mean marker interval of 18.68 cM, and only 212 markers were mapped, thus limiting its application in further QTL localization and genome assembly (Lv et al., 2017).

Herein, we randomly selected 129 G_1_ offspring from one full-sib family of *S. paramamosain* for SNP mining and genotyping using SLAF-seq method. A high-density genetic linkage map (0.81 cM average marker interval) of *S. paramamosain* with 16,701 SNP markers was successfully constructed, spanning a total of 5,996.66 cM in 49 linkage groups (LGs). The inclusion of 8 female-specific SNP markers enabled the identification of two QTL regions on LG32 that were linked with sex determination. Based on the growth-related traits measurements, 27 quantitative trait loci (QTLs) of growth-related traits were also identified. In addition, growth-related traits associated SNP markers were detected as well. Moreover, 11 and 2 candidate genes were identified within the QTL regions of sex and growth-related traits, respectively. This study provides novel insights into the sex determination system and growth performance of mud crab and in other related crustacean species.

## Materials and Methods

### Ethics Statement

The animal experimental procedures used in this study were approved and conducted in strict accordance with the recommendations in the *Guide for the Care and use of Laboratory Animals* outlined by the Institutional Animal Care and Use Ethics Committee of Shantou University and the National Institutes of Health guide for the care and use of Laboratory animals (NIH Publications No. 8023, revised 1978).

### Mapping Population Collection and DNA Extraction

The mud crab *S. paramamosain* is a common aquaculture species in southeastern coastal areas of China. The parents were cultured and mated in a pond, and the full-sib G1 family were produced in a hatchery located at Raoping, China. The offspring were artificially reared with commercial feed and low-value fishes to maturity in the same pond. A total of 129 progenies (63 males; 66 females) was randomly collected 4 months post-hatch for linkage mapping analysis and growth traits measurement. The measured growth traits include: carapace length (CL), carapace width (CW), internal carapace width (ICW), carapace frontal width (CFW), abdomen width (AW), body height (BH), carapace width at spine 8 (CWS8), distance between frontal median spine (DFMS), distance between frontal lateral spine (DFLS), distance between lateral spine 1 (DLS1), distance between lateral spine 2 (DLS2), fixed finger length of the cheliped (FFLC), fixed finger width of the cheliped (FFWC), fixed finger height of the cheliped (FFHC), meropodite length of pereopod 1 (MLP1), meropodite length of pereopod 2 (MLP2), meropodite length of pereopod 3 (MLP3), dactyl length of pereiopod 4 (DLP4), dactyl width of pereiopod 4 (DWP4), and body weight (BW) (Ma et al., 2013; Fazhan et al., 2017b). The 19 morphological traits were measured to the nearest 0.01 mm using standard Vernier caliper. BW was measured to an accuracy of 0.01 g with a digital electronic balance. Sex of each crab was determined based on its gonad morphology after dissection (Quinitio et al., 2007; Waiho et al., 2017). Genomic DNA from muscle tissues of the right cheliped of the maternal parent and 129 progenies were extracted using conventional CTAB DNA extraction method. The extracted DNA’s quantity and quality were checked using Nanodrop 1000 spectrophotometer (Thermo Scientific, Wilmington, DE, United States) and agarose gel electrophoresis (1% concentration), respectively, before storing at -80°C until further analysis.

### SLAF-Seq Library Construction and High-Throughput Sequencing

Specific-locus amplified fragment sequencing (SLAF-seq) libraries were constructed and sequenced based on the method of Sun et al. (2013) with slight modification. Preliminary marker discovery was stimulated *in silico* based on the reference genome of *E. sinensis*. Restriction endonucleases HaeIII and Hpy166II (New England Biolabs, NEB) were selected and used to digest the genomic DNA of *S. paramamosain* G_1_ population. Subsequently, a single nucleotide (A) overhang was added to the digested fragments using Klenov (3′ → 5′ exo^-^) (NEB) and dATP. Both steps were incubated at 37°C. Duplex tag-labeled sequencing adapters (PAGE-purified, Life Technologies, United States) were then ligated to the A-tailed fragments using T4 DNA ligase. The diluted restriction-ligation DNA products were then subjected to Polymerase Chain Reaction (PCR) using Q5^®^ High-Fidelity DNA Polymerase and specific primers (forward: 5′-AATGATACGGCGACCACCGA-3′; reverse:5′-CAAGCAGAAGACGGCATACG-3′) (Life Technologies) and subsequently purified using Agencourt AMPure XP beads (Beckman Coulter, High Wycombe, United Kingdom). Purified products were then pooled and separated by 2% agarose gel electrophoresis. Fragments in the range of 314–414 bp (with indexes and adaptors) were excised and purified using QIAquick gel extraction kit (Qiagen, Germany). After purification, paired-end 125 bp sequencing was performed on the Illumina HiSeq 2500 platform (Illumina Inc., CA, United States). Asian rice *Oryza sativa japonica* (genome size 382 M) was used as control and subjected to the same sequencing procedure to assess the accuracy of library construction.

### Sequencing Data Grouping and Genotyping

The discovery and genotyping of SLAF markers were conducted based on Sun et al. (2013). Raw reads were sorted to each progeny according to the duplex barcode sequences after the removal of low-quality reads (reads with quality score of less than 20e, with e represents base sequencing error rate). Next, barcodes and terminal 5-bp positions were trimmed from each raw read. Sequences that were mapped to the same position with high similarity (>95% identity) were defined as a SLAF locus (Zhang et al., 2015). SNP loci of each SLAF locus were detected between parents using Genome Analysis Toolkit (GATK). To ensure the accuracy, SNP calling was also carried out using SAMtools. Only variants called out by both algorithms (GATK and SAMtools) were considered as SNPs. Further, GATK with default parameters was used for the removal of duplicated reads, realignment of reads around insertions/deletions, and recalibration of base quality. Reads with quality by depth (QD) score < 2.0, mapping quality (MQ) of <40 and Fisher Strand (FS) score of >60 were filtered out. All polymorphic SNPs were genotyped for consistency with the parental and offspring SNP loci. Genotype scoring based on Bayesian approach was performed to ensure genotyping quality (Sun et al., 2013). To identify polymorphic SNPs, firstly, SNPs with average depth of less than 10× in parent and 4× in offspring were filtered out. Next, only SNPs with at least 90% frequency among all offspring were selected. Lastly, markers with significant segregation distortion (based on Chi-square test, *P* < 0.05) were excluded from map construction but added later as accessory markers. Based on their SNP genotypes, five segregation patterns (ab × cd, ef × eg, hk × hk, lm × ll, nn × np) were used to construct the full-sib family linkage map. The paternal genotype was deduced based on the maternal and offspring genotypes. Further, 13 SNP markers were selected for validation of the accuracy of genotyping (Supplementary Table S1).

### Linkage Map Construction

Eight female-specific SNP markers were added (Supplementary Table S2) in addition to the high-quality SNP markers generated. Marker loci were first partitioned into LGs. The markers’ robustness for each LGs were validated by filtering out markers with modified logarithm of odds (MLOD) scores of less than 6. Additionally, 8 sex-specific SNP markers that were heterozygous in female *S. paramamosain* were added to the selected markers for genetic linkage map construction. HighMap strategy was employed for the construction of a high-density and high-quality map (Liu et al., 2014). Firstly, linkage phases were inferred based on recombinant frequencies and LOD scores estimated by two-point analysis. Then, the process of marker ordering was conducted by combining the enhanced gibbs sampling, spatial sampling and simulated annealing algorithms (GSS) (Jansen et al., 2001; Van Ooijen, 2011). After several cycles, a stable map order was obtained. A subset of currently unmapped markers was then added to the previous sample with decreased sample radius for subsequent mapping. The process was repeated until all markers were mapped accordingly. SMOOTH strategy and *k*-nearest neighbor algorithm were used to correct errors based on parental contribution of genotypes and to impute missing genotypes, respectively (van Os et al., 2005; Huang et al., 2012). Skewed markers were subsequently inserted into the linkage map via multipoint method of maximum likelihood. The sex-specific maps were constructed based on heterozygous markers in either parents whereas the consensus map was built by integrating the maps of both parents via anchor markers. Kosambi mapping function was then used to estimate map distances (Kosambi, 1943). The expected genome size (*G*_e_) was calculated using the formula:

$$\begin{array}{r} \begin{array}{lll} \begin{array}{r} Ge \end{array} & = & (Ge1+Ge2)/2\\ With\;Ge1 & = & \sum (LGOL+2s),Ge2\\ & = & \sum [LGOL\times ((m+1)/(m-1] \end{array} \end{array}$$

where LG_OL_ is the observed length of linkage group, *s* represents the average marker interval and *m* is the number of markers in each linkage group (Chakravarti et al., 1991; Ma et al., 2016). The estimated genome coverage was then calculated as the percentage of observed genome size divided by *G*_e_ (Liao et al., 2007; Jones et al., 2013; Ma et al., 2016). The ratio of female: male recombination rate was calculated using two methods, (1) based on the full length of each LG, and (2) based on the length of shared markers between female and male linkage maps (specifically for LG32).

### QTL Analysis and Candidate Genes Identification

The QTL analysis was conducted using the R/QTL package (Broman and Sen, 2009) to link phenotypic trait measurements with genotypic data in an attempt to uncover the genetic basis of variation in the measured traits (Kearsey, 1998). The phenotypic sex was treated as a binary trait (0 for females and 1 for males). Composite Interval Mapping (CIM) was performed for each trait. The LOD threshold for each data set was acquired based on permutation test (1,000 permutations, *P* < 0.05). We then identified their candidate genes based on the obtained QTL of sex and growth-related traits. In brief, SNPs on QTLs were grouped as SLAF marker based on the initial SLAF sequencing. They were then compared to the assembled and annotated gonadal transcriptome sequences of mature *S. paramamosain* (GenBank ID: SRR5387739 and SRR5387741) by NCBI^1^ Blast+. The parameters were set as *e*-value < 1e^-05^, identities > 90% and sequence length of alignment > 80 bp. The candidate genes were obtained according to the annotation of the transcriptome sequence.

### Statistical Analyses

All statistical analyses were conducted using IBM SPSS Statistic ver. 20 and Microsoft Excel 2016. Pearson’s correlation test was conducted between every two traits to determine their strength of association. Generalized linear model (GLM) was used to evaluate the association between markers of different genotypes and the expected growth-related traits derived from QTL analysis, with the genotype as explanatory variable and growth-related traits as dependent variables. Subsequent Student–Newman–Keuls (SNK) method was conducted if significant differences among genotypes occurred. All results were statistically significant at *P* < 0.05.

## Results

### SLAF Sequencing Summary

Before the construction of the sequencing library, restriction enzymes were chosen based on the predicted number of SNPs and length of produced fragments. With an insert size range of 314–414 bp, a combination of restriction enzymes HaeIII and Hpy166II was expected to produce the highest number of SNP markers (227,798) and to achieve the 93.49% digestion efficiency in the control sample. Libraries construction and sequencing of parent and 129 progenies using HaeIII and Hpy166II generated 731.37 M high quality pair-end reads, with Q30 percentage of 93.92% and GC percentage of 41.78% (Table 1). Of the five major patterns that could be used in linkage map construction, nn × np was the major pattern (43.44%), in contrast, ab × cd accounted for only 0.01% of the total SNP number (Supplementary Figure S1). After filtering markers with MLOD values of less than 6, 16,693 out of 17,246 markers were selected for subsequent genetic linkage map construction (Table 1).

**Table 1:** SLAF-seq data statistics in *Scylla paramamosain*.

Category	
**Total reads**	
Maternal parent reads (Q30 percentage, %; GC percentage, %)	25,788,354 (94.38; 41.85)
Offspring reads (Q30 percentage, %; GC percentage, %)	5,469,612 (93.92; 41.78)
Number of total reads (Q30 percentage, %; GC percentage, %)	731,368,260 (93.92; 41.78)
**SNPs**	275,876
Total SNPs	75,461
Markers lacking in either parent	56,902
Parental markers depth less than 4×	7,567
Number of Non-polymorphic markers in parent	135,946
Remaining markers	131,737
SNP used for polymorphic marker determination (excluding aa × bb)	
SNPs with low depth	51,217
SNPs with less than 90% frequency	59,156
SNPs with segregation distortion	4,118
Polymorphic SNPs	17,246
SNPs with low MLOD	553
Useful SNPs	16,693
**Sequencing depth**	
Total marker number in maternal parent (MLOD > 6)	16,693
Total sequencing depth in maternal parent	1,702,737
Average depth in maternal parent	102×
Total marker number in maternal parent (MLOD > 6)	16,638
Total sequencing depth in offspring	376,898
Average depth in offspring	22.65×

### Construction of Genetic Linkage Maps

**FIGURE 1:**
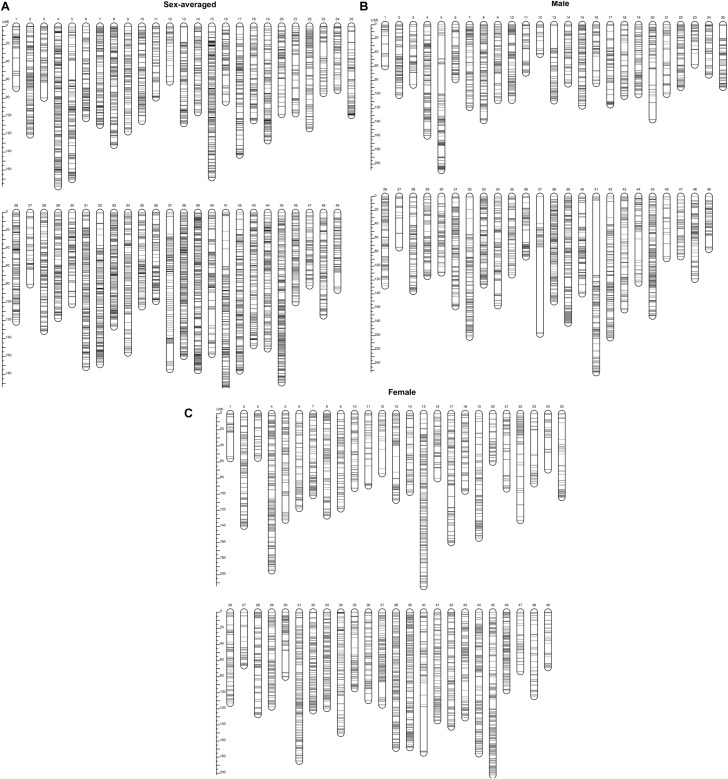
The genetic linkage maps of **(A)** sex-average, **(B)** male, and **(C)** female *Scylla paramamosain*.

Based on a pseudo-testcross strategy, a genetic linkage map containing 49 LGs with 16,701 markers were constructed with a high 99.95% individual integrity value (Table 2). The total length of male and female linkage maps were 5,877.71 and 5,790.08 cM, respectively. These two sex-specific linkage maps were integrated into a sex-averaged map (Figure 1) that spanned 5,996.66 cM with an average marker interval of 0.81 cM (Table 2). The detail information of the genetic linkage map for female, male and sex-averaged were shown in Supplementary Table S3. The estimated genome size was 6,004.58 cM for male, 5,907.38 cM for female, and 6,076.28 cM for the sex-averaged. Based on the ratio of the observed and estimated sizes, the genome coverage of the male, female and sex-average maps were 97.89, 98.01, and 98.69%, respectively.

**Table 2:** Summary of *Scylla paramamosain* linkage maps.

	Sex-averaged map	Male map	Female map
Number of linkage group	49	49	49
Number of SNP markers	16,701	9,359	9,742
Number of SLAF markers	8,906	5,827	6,023
Total length of linkage group (cM)	5,996.66	5,877.71	5,790.08
Minimum length of linkage group (cM)	61.73	41.89	54.96
Maximum length of linkage group (cM)	194.24	252.47	214.36
Average SLAF marker interval (cM)	0.81	1.29	1.20

### Segregation Distortion

Of the 16,701 markers mapped on the genetic linkage map, only 187 were skewed markers (Supplementary Figure S2), representing a mere rate of 1.12%. These skewed markers were distributed in 26 out of 49 LGs, with LG34 and LG5 exhibited highest number of skewed markers, 28 and 25, respectively. Including LG34 and LG5, only 14.29% of the LGs had more than 10 skewed markers. The number of skewed markers in these 26 linkage groups ranged from 1 to 28.

### QTL Mapping for Sex and Growth-Related Traits

**FIGURE 2:**
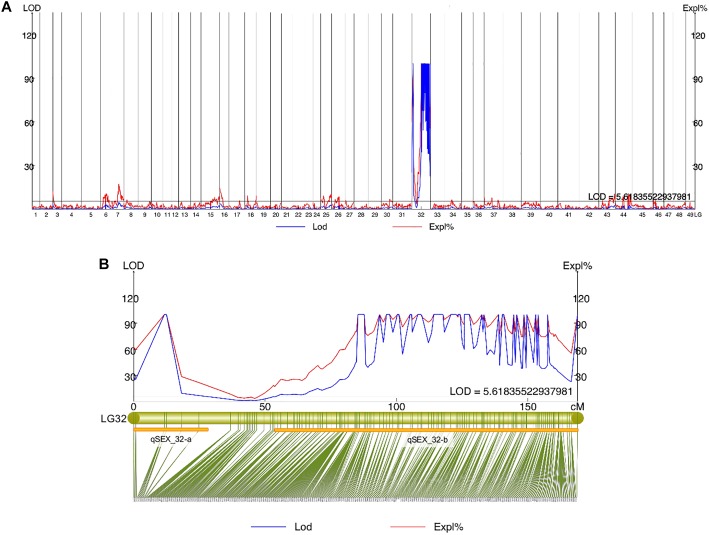
**(A)** Sex QTL mapping and association analysis in *Scylla paramamosain* in all linkage groups. **(B)** The location of two QTLs (qSEX_32-a and qSEX_32-b) on LG32, their corresponding LOD and PVE values, and their respective markers. All eight female-specific SNP markers were in qSEX_32-b.

Quantitative trait locis for phenotypic sex trait were exclusively found on LG32, with 516 markers distributed in two QTL regions and covered approximately 86.72% of the 168.88 cM LG length (Figure 2 and Table 3). Specifically, the 8 added female-specific SNP markers showed complete linkage with phenotypic sex and were in the region of 109.70–123.43 cM of the sex-averaged linkage map, 79.69–87.55 cM in the female linkage map and absent in the male linkage map (Supplementary Tables S5, S6), with an average proportion of phenotypic variation explained by these 8 female-specific SNP markers to be more than 99% (Supplementary Table S4). Hence, the presence of 8 female-specific SNP markers in female but not male linkage map strongly favor female over male heterogamety in *S. paramamosain*, i.e., a ZW/ZZ sex determination system. Further, the observed segregation patterns to those expected for female-specific SNP markers under the assumption of a ZW/ZZ or XY/XX system were compared (Table 4). All 8 female-specific SNP markers exhibited segregation pattern 1 expected under female heterogamety and none segregated according to patterns 6, 7, or 8, expected under the assumption of male heterogamety. Additionally, 7 estimated markers sharing the same location with the 8 female-specific SNP markers from the linkage map were identified, and when tabulated according to their segregation patterns, 6 showed the pattern 1 (Table 4). Thus, this further highlights the involvement of this region in LG32 as possible sex determination region. The male-skewed female: male recombination rate ratios of all markers (recombination ratio = 0.61) (Table 5) and shared markers (recombination ratio = 0.64) (not shown) on LG32 reflect the lower recombination rate of female. Thus, coupled with the linkage data and the linkage patterns observed in the female-specific SNP markers, the skewed recombination rate ratios firmly establish female heterogamety in *S. paramamosain*.

**Table 3:** Quantitative trait loci (QTLs) for sex and growth-related traits of *Scylla paramamosain*.

Trait	LG	QTL name	LOD threshold	Position (cM)		Number of SNP markers	LOD	PVE (%)	Max PVE (%)
				Start	End				
Sex	32	qSEX_32-a	5.62	0.00	24.85	18	16.18	72.45	100
		qSEX_32-b		53.25	168.88	498	15960.76	75.16	100
CL	46	qCL_46-a	3	26.39	33.74	43	3.81	7.55	14.1
CW	46	qCW_46-a	3	26.39	33.74	43	3.96	7.95	14.4
ICW	46	qICW_46-a	3	26.39	33.74	43	3.87	7.85	14
CFW	46	qCFW_46-a	3	26.39	33.74	43	3.89	7.82	14
AW	46	qAW_46-a	3	26.39	33.74	43	4.03	7.82	15.1
BH	46	qBH_46-a	3	26.39	33.74	43	3.78	7.85	13.9
CWS8	46	qCWS8_46-a	3	26.39	33.74	43	3.98	8.04	14.5
DFMS	17	qDFMS_46-a	3	9.20	12.14	9	3.18	10.49	10.9
DFLS	40	qDFLS_46-a	3	0.00	6.47	19	3.56	11.95	13.4
DLS1	40	qDLS1_40-a	3	129.16	129.16	2	3.05	9	9
		qDLS1_40-b		135.81	135.81	3	3.10	8.9	8.9
		qDLS1_40-c		143.32	144.91	8	3.06	10.44	10.9
		qDLS1_40-d		154.57	157.45	7	3.10	10.5	10.5
	46	qDLS1_46-a	3	30.06	30.06	14	3.20	5.8	10.8
DLS2	40	qDLS2_40-a	3	0.00	0.00	5	3.10	10.5	10.5
	46	qDLS2_46-a	3	28.84	32.68	39	3.50	7.07	12.5
FFLC	46	qFFLC_46-a	3	30.06	30.84	18	3.03	6.75	10.3
FFWC	46	qFFWC_46-a	3	25.18	32.68	41	3.64	8.78	12.7
FFHC	46	qFFHC_46-a	3	26.39	31.62	38	3.32	6.3	11.8
MLP1	46	qMLP1_46-a	3	28.84	31.23	28	3.41	6.19	11.9
MLP2	46	qMLP2_46-a	3	30.84	30.84	4	3.08	10.4	10.4
MLP3	46	qMLP3_46-a	3	30.84	31.23	7	3.17	10.7	11
DLP4	40	qDLP4_40-a	3	0.00	1.58	6	3.23	10.9	11
	46	qDLP4_46-a	3	7.45	7.45	3	3.21	9.37	9.4
		qDLP4_46-b		28.84	32.68	39	3.34	7.91	12.6
DWP4	46	qDWP4_46-a	3	30.06	31.23	21	3.14	7.57	10.9
BW	46	qBW_46-a	4.80	30.06	30.06	14	4.80	8.9	15.8

**Table 4:** Expected and observed segregation patterns for female-specific SNP markers (*n* = 8) and estimated markers sharing the same loci with female-specific SNP markers (*n* = 7) under the assumption of either a ZW/ZZ or a XY/XX sex determination system (according to Staelens et al., 2008).

Pattern	Dam genotype	Sire genotype	Genotype of female offspring	Genotype of male offspring	Number of observation in F1	
					Female-specific SNP markers	Estimated markers
ZW/ZZ						
1	*Aa*	*aa*	*Aa*	*aa*	8	6
2	*aA*	*aa*	*aa*	*Aa*	0	0
3	*aA*	*AA*	*aA*	*AA*	0	0
4	aA	aA	Aa or aa	AA or Aa	NI	NI
5	aa	Aa	Aa or aa	Aa or aa	NI	NI
XY/XX						
6	*aa*	*aA*	*aa*	*Aa*	0	0
7	*aa*	*Aa*	*Aa*	*aa*	0	0
8	*AA*	*Aa*	*AA*	*Aa*	0	0
9	Aa	Aa	AA or Aa	Aa or aa	NI	NI
10	Aa	aa	Aa or aa	Aa or aa	NI	NI

*ZW/ZZ, patterns 1–5; XY/XX, patterns 6–10; NI, not informative; Segregation patterns unique for the two sex determination systems are in italics; ‘A’ and ‘a’ are symbols and do not represent allelic dominance. One estimated marker (marker 19218) exhibited pattern 4*.

**Table 5:** The SNP marker numbers and recombination rates (all markers) of each LG of *Scylla paramamosain*.

LG	SNP marker numbers					Recombination rates				
	Female	Male	Female only	Male only	Shared	Female (cM)	Male (cM)	Female and male ratio
1	63	43	50	30	13	55.48	59.66	0.93
2	218	209	154	145	64	139.47	100.83	1.38
3	81	93	62	74	19	54.96	86.1	0.64
4	428	283	348	203	80	194.85	159.24	1.22
5	219	267	166	214	53	131.85	208.66	0.63
6	121	144	95	118	26	117.36	78.39	1.50
7	223	168	156	101	67	101.37	118.21	0.86
8	216	213	168	165	48	126.64	136.77	0.93
9	184	203	127	146	57	117.88	108.33	1.09
10	114	272	54	212	60	92.39	107.92	0.86
11	58	92	43	77	15	89.22	68.78	1.30
12	42	39	28	25	14	74.25	41.89	1.77
13	172	186	131	145	41	107.22	108.71	0.99
14	98	100	70	72	28	97.35	84.43	1.15
15	432	326	318	212	114	214.36	116.14	1.85
16	191	129	139	77	52	80.49	83.78	0.96
17	297	238	241	182	56	159.71	114.42	1.40
18	147	156	108	117	39	95.85	102.25	0.94
19	171	203	130	162	41	154.31	99.84	1.55
20	155	173	129	147	26	59.85	135.89	0.44
21	97	113	82	98	15	93.05	99.24	0.94
22	140	212	104	176	36	132.35	89.38	1.48
23	74	71	55	52	19	86.81	57.34	1.51
24	67	108	50	91	17	69.48	71.61	0.97
25	122	165	89	132	33	103.69	89.33	1.16
26	165	203	112	150	53	112.85	127.95	0.88
27	60	39	45	24	15	66.24	73.17	0.91
28	255	216	192	153	63	126.88	135.88	0.93
29	214	158	159	103	55	117.53	114.32	1.03
30	162	215	115	168	47	80.36	109.26	0.74
31	338	250	267	179	71	184.79	157.73	1.17
32	338	350	245	257	93	122.17	201.5	0.61
33	309	328	244	263	65	119.32	126.55	0.94
34	227	186	138	97	89	150.26	156.34	0.96
35	151	136	104	89	47	94.6	112.04	0.84
36	146	186	103	143	43	109.38	86.28	1.27
37	267	77	240	50	27	115.11	197.14	0.58
38	384	394	290	300	94	168.71	150.81	1.12
39	417	443	312	338	105	167.9	181.37	0.93
40	150	248	100	198	50	174.62	139.36	1.25
41	267	285	194	212	73	134.44	252.47	0.53
42	248	285	192	229	56	142.35	202.44	0.70
43	226	189	173	136	53	130.89	162.19	0.81
44	344	158	286	100	58	175.63	123.91	1.42
45	412	403	319	310	93	202.19	171.3	1.18
46	293	30	274	11	19	97.15	88.64	1.10
47	90	107	65	82	25	73.51	86	0.85
48	80	158	38	116	42	104.4	118.42	0.88
49	69	109	38	78	31	68.56	75.5	0.91

**FIGURE 3:**
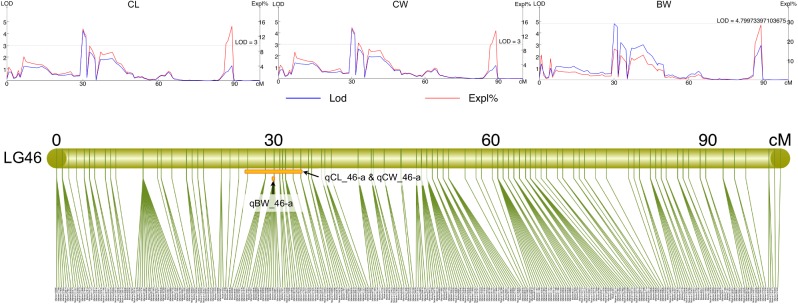
The LOD and PVE values, and the QTL location of CL (qCL_46-a), CW (qCW_46-a), and BW (qBW_46-a) on LG46.

All measured growth traits and BW of the 129 progenies (Supplementary Table S7) showed significant correlation between one another (*P* < 0.001) (Supplementary Table S8). Using the Composite Interval Mapping Method, a total of 27 significant QTLs for growth-related traits were detected (Table 3). Of the 20 growth traits, the QTLs for 18 traits, including some economically important traits such as CL, CW, and BW were found on LG46, with most SNPs being found in the narrow region of 25.18–33.74 cM (Table 3 and Supplementary Table S4). Thus, this region was postulated to be the candidate genomic region involved in the growth regulation of *S. paramamosain*. The PVE values of all growth-related traits were in the range of 5.8% to 11.95%, with maximum PVE values lie between 8.9 and 15.8% (Table 3). Interestingly, the QTLs for CL, CW, ICW, CFW, AW, BH, and CWS8 shared the same 43 SNPs on LG46 from 26.39 to 33.74 cM, highlighting the relatedness among these growth-related traits. The full list of markers corresponding to each trait is available in Supplementary Table S4. Specifically looking at the economically important traits, the QTLs of CL and CW both occupy the same region, 26.39 to 33.74 cM, whereas that of BW was on 30.06 CM of LG46 (Supplementary Table S4 and Figure 3). The QTLs of these three economically important traits recorded high maximum PVE values, with that of CL, CW and BW being 14.1, 14.4, and 15.8%, respectively (Table 3), and 14 markers were shared among them (Supplementary Table S4).

Of the 166 SNP markers within the QTLs of sex and growth-related traits, 23 (16 were the same marker on different QTLs) were successfully annotated to the transcriptome assembly of *S. paramamosain* using Blast+ (Table 6 and Supplementary Table S9). Eleven candidate genes were identified from a single sex QTL on LG32 (qSEX_32-b), including 26S proteasome non-ATPase regulatory subunit 3 (*PSMD3*), RNA polymerase II subunit A C-terminal domain phosphatase (*CTDP1*), low density lipoprotein receptor-related protein 2 (*LRP2*) and protein FAM126B (*FAM126B*). Due to the overlapping QTL regions on LG46 for most growth-related traits, the same SNP marker was on the 16 growth-related traits’ QTLs (Supplementary Table S9). This marker (marker 2388) showed significant similarity with an unigene that is annotated to multidrug resistance-associated protein 4 (*MRP4*) and cystic fibrosis transmembrane conductance regulator (*CFTR*).

**Table 6:** Summary of candidate genes for sex and growth traits in *Scylla paramamosain.*

Traits	LG	QTL name	SNP ID	Unigene ID	Candidate genes
Sex	32	qSEX_32-b	Marker1101	TR40511| c0_g1_i1	26S proteasome non-ATPase regulatory subunit 3 (*PSMD3*); PCI domain-containing protein 2 homolog (*PCID2*); COP9 signalosome complex subunit 3 (*CSN3*)
			Marker3514	TR24805| c0_g1_i2	RNA polymerase II subunit A C-terminal domain phosphatase (*CTDP1*)
			Marker21423	TR31065| c0_g1_i1	Zinc finger CCCH domain-containing protein 4 (*ZC3H4*); E3 ubiquitin-protein ligase makorin-1 (*MKRN1*); mRNA 3′-end-processing protein YTH1 (*YTH1*)
			Marker131703	TR63327| c0_g1_i2	low density lipoprotein receptor-related protein 2 (*LRP2*)
			Marker11278	TR33359| c0_g1_i1	Protein FAM126B (*FAM126B*)
			Marker135124	TR21815| c0_g1_i1	UBX domain-containing protein 1 (*UBXN1*)
			Marker134510	TR52925| c0_g1_i1	Retrovirus-related Pol polyprotein from type-2 retrotransposable element R2DM (*POL*)
CL	46	qCL_46-a	Marker2388	TR75260| c0_g1_i1	Multidrug resistance-associated protein 4 (*MRP4*); Cystic fibrosis transmembrane conductance regulator (*CFTR*)
CW	46	qCW_46-a			
ICW	46	qICW_46-a			
CFW	46	qCFW_46-a			
AW	46	qAW_46-a			
BH	46	qBH_46-a			
CWS8	46	qCWS8_46-a			
DLS1	46	qDLS1_46-a			
DLS2	46	qDLS2_46-a			
FFLC	46	qFFLC_46-a			
FFWC	46	qFFWC_46-a			
FFHC	46	qFFHC_46-a			
MLP1	46	qMLP1_46-a			
DLP4	46	qDLP4_46-b			
DWP4	46	qDWP4_46-a			
BW	46	qBW_46-a			

*Unigene ID, the ID of unigene from the gonadal transcriptome data of mature S. paramamosain (GenBank ID: SRR5387739 and SRR5387741).*

### Association Between SNP Markers and Growth-Related Traits

Of the 95 SNP markers distributed in 27 QTLs of growth-related traits, more than half (67) showed significant association with at least one growth-related traits, and all 20 growth-related traits had at least one associated marker (Supplementary Table S10). Further, four markers (23029, 3846, 7391, and 8848) were significantly associated with 10–16 growth-related traits (*P* < 0.05) (Supplementary Table S10). Interestingly, at three markers (i.e., 23029, 3846, and 7391), individuals with genotype hk were of larger size in terms of CL and CW compared to those of genotype hh and kk. Similar pattern was also observed in the association between marker 3846 and BW, where hk-genotype individuals were significantly heavier compared to the other two genotypes. The higher average values of CL and CW in individuals with genotype hk at markers 3846 and 7391 indicate that genotype hk at these two markers are better in selecting CL and CW compared to genotype hk at markers 23029 and 8848. Unlike individuals with genotype hk × hk, those with genotype ll × lm and nn × np did not show any significant association with economically important growth traits, i.e., CL, CW, and BW (Supplementary Table S10).

## Discussion

### SLAF-Seq in *Scylla paramamosain*

SLAF-seq is an improved version of the common RAD sequencing, a reduced representation sequencing technology which enables large-scale genotyping and calling of SNPs based on the sampling of genome-wide enzyme loci developed via next-generation sequencing (Sun et al., 2013). The use of restriction enzymes to generate myriads of DNA fragments is the basis for RAD sequencing and its derivatives, including SLAF-seq. In the present study, after sequencing, the number of obtained SNPs was in accordance with the estimated *in silico* digestion simulation (275,876 SNPs were obtained, and 227,798 SNPs were estimated), indicating the high consistency between simulated and actual digestion using the predicted restriction enzymes, and the suitability of HaeIII and Hpy166II for SLAF-seq of *S. paramamosain*.

Of the 275,876 SNPs, a total of 17,246 were polymorphic in the mapping population. The high sequencing depth in parent (102×) and offspring (average 22.65×) guaranteed the accuracy of SLAF-seq in the current study. Due to the ability to obtain large numbers of high-quality SNPs, SLAF-seq has been utilized in organisms with and without a reference genome sequence (Sun et al., 2013), including other brachyuran species such as *E. sinensis* (Qiu et al., 2017) and *P. trituberculatus* (Lv et al., 2017). A high success rate of 96.79% (16,693 out of 17,246 SNPs) polymorphic markers was used in the *S. paramamosain* linkage map construction. The high number of polymorphic markers suggests high heterozygosity and complexity of the *S. paramamosain* genome. After the addition of 8 female-specific SNP markers, all 16,701 SNPs (100%) were successfully assigned into the linkage map.

The genome of crustacean is very repetitive (Song et al., 2016; Yuan et al., 2017; Zhang et al., 2019). Thus, removing reads with multiple targets in genome is beneficial for increasing the quality of genetic linkage map. In our preliminary genome survey of *S. paramamosain*, the genome repetitive rate was 60.8% (unpublished data). It is believed that with the availability of *S. paramamosain* draft genome in future, we would be able to enhance the quality of the current genetic linkage map through the prediction of reads’ targets and removal of multiple-target reads.

### Linkage Mapping

The constructed high-density genetic linkage map of *S. paramamosain* based on 16,701 SNP markers has 49 LGs and spanned a total distance of 5,996.66 cM. The current linkage map is the second genetic linkage map, the first high-quality linkage map of *S. paramamosain*. Compared to the first genetic linkage map of 2,746.4 cM in length and constructed based on a combination of 60 microsatellites and 152 AFLP markers (Ma et al., 2016), the current genetic linkage map is of higher density and comprises almost 78-fold more markers. The larger genetic linkage map length found in our study is expected as the increase in marker density (Ball et al., 2010) will increase the power of detecting recombination (more chance to map markers into marker absence regions), enlarging the length of the genetic linkage map accordingly as more recombination events could be recognized. Low marker densities may underestimate map length (Slate, 2008). Unlike the high-density linkage maps now, previous generations of linkage maps were conducted with low number of markers, resulting in a smaller genome coverage. Similar increase in map length (almost doubled) due to the increase in marker densities were also reported in the Chinese mitten crab (Cui et al., 2015; Qiu et al., 2016, 2017) and turbot (Ruan et al., 2010; Bouza et al., 2012; Wang W. et al., 2015). Another reason might be related to the number of chromosomes. The number of chromosomes (*n* = 49) in *S. paramamosain* is high (Chen et al., 2004), and with the low marker numbers used in previous linkage map construction (Ma et al., 2016), some chromosome might not be mapped, resulting in the missing information of the whole chromosomal genome and consequently smaller genome coverage. The high genome coverage (>97%) and small average marker interval (0.81 cM) reflect the high density and quality of the constructed genetic linkage map (Lv et al., 2017; Qiu et al., 2017). The much higher resolution of the SNP-based linkage map facilitates more accurate and detailed QTL mapping, provides more anchor points for whole genome sequences assembly as well as to serve as new chromosome framework for comparative genomic studies with other closely related organisms.

The number of LGs of our current constructed genetic linkage map is consistent with the reported number of haploid chromosomes of *S. paramamosain* (*n* = 49) by Chen et al. (2004) (noted that although Chen et al., 2004 described the investigated species as *Scylla serrata*, it should be *S. paramamosain* because of the wrong nomenclature). The higher number of LGs (*n* = 65) from previously constructed genetic linkage map (Ma et al., 2016) might be due to the weak linkage between markers. Additionally, lower marker density (only 212 markers) used in the previous study may be another reason leading to more LGs. When marker numbers are low, huge gaps exist between markers, thus more linkage groups will be predicted (Da Costa E Silva et al., 2007, Da Costa et al., 2012; Tao et al., 2017). Similar reduction of the estimated number of LGs when comparing first generation with second generation linkage maps was also observed in other organisms, such as in the common carp *Cyprinus carpio* [from 64 LGs (Cheng et al., 2010) to 50 LGs (Peng et al., 2016)] and pear *Pyrus* spp. [from 18 LGs (Iketani et al., 2001) to 17 LGs (Wu et al., 2014)]. Thus, this improved linkage map, with 49 LGs and 16,701 markers, is presently the densest mud crab linkage map.

### Segregation Distortion

Segregation distortion is the deviation of the segregation ratio of a locus from the expected Mendelian ratio. Such distortion is common in linkage map studies and genome analysis, and the proportion of skewed markers varied among species (Wang et al., 2016). The percentage of skewed markers found in this study (1.12%), however, is comparatively lower than that of previous study (32.10%). The low average frequency of skewed markers in our linkage map could be due to several internal molecular factors, including zygotic viability selection, genes duplication, transposable elements, and unusual meiotic segregation distortion. Additionally, segregation distortion has also been reported to be caused by factors such as small population size and types of genotyping markers used, as in the case of our previous study (Ma et al., 2016). The genetic linkage maps of *E. sinensis* had a skewed marker percentage of 15.72% (Qiu et al., 2017) when constructed based on a combination of SNP and SSR markers, and 16.76% when genotyped using only SSR markers (Qiu et al., 2016). The high degree of linkage (average = 98.88%) between adjacent markers of each LGs suggests that segregation distortion does not substantially impact QTL mapping, instead, incorporation of these skewed markers during the construction of genetic linkage maps could enhance genome coverage and improve the detection of linked QTLs (Xu, 2008; Qiu et al., 2017).

### QTL for Sex

Sex determination is an essential part of reproduction and holds significant importance in genome evolution (Cui et al., 2015). The sex determination system in crustaceans is controversial, with early karyotyping studies suggest a XY–XX sex determination system (Niiyama, 1938; Lécher et al., 1995), but recent analysis of genetic linkage map revealed an ZW/ZZ sex determination system in Chinese mitten crab *E. sinensis* (Cui et al., 2015), brine shrimp *Artemia franciscana* (De Vos et al., 2013) and black tiger shrimp *Penaeus monodon* (Staelens et al., 2008). Coupled with the recent discovery of several female-specific SNP markers in *S. paramamosain* (Shi et al., 2018), we believe that female heterogametic sex determination system (ZW/ZZ) is one of sex determination systems in crustaceans. In the current study, the phenotypic sex trait mapped 516 markers to a single LG–LG32 (168.88 cM). The high coverage of these markers (approximately 86.72%) on LG32 suggests that the sex determination system of *S. paramamosain* is polygenic but sex determining QTLs are located on the same LG, unlike in some fish species, with sex-determining QTLs being spread in several LGs (Palaiokostas et al., 2015b). The presence of female-specific SNP markers exclusively on female linkage map, their near 100% PVE values, their segregation patterns that comply to that of ZW/ZZ system (Staelens et al., 2008), and their lower recombination rate on LG32 strongly imply that the sex determination system of *S. paramamosain* follows a ZW/ZZ system, with LG32 as the putative sex chromosome. These findings serve as solid foundation for future sex-manipulation of *S. paramamosain*. Future studies involving triploidy induction by retention of the second polar body is recommended to investigate the tendency of feminization in *S. paramamosain* larvae and to further validate the suggested ZW/ZZ system (Sellars et al., 2010), with sex ratio of induced triploids skew toward female is expected. Similar triploidy induction method was also successfully applied as a validation strategy of the suggested ZW/ZZ sex determination system in *E. sinensis* (Cui et al., 2015). The 6 estimated markers that shared the same loci with female-specific SNP markers and exhibited similar female heterogamety pattern should be further investigated as well. These female-specific markers could be useful in future genetic analysis of the sex chromosome of *S. paramamosain* and other *Scylla* species (Jairin et al., 2013). With the completion of the *S. paramamosain* whole genome sequencing in the future, this region containing female-specific SNP markers should provide useful insights into the genes involved in sex determination mechanism of this economically important crustacean species.

To further identify potential genes within QTL for sex, we compared the markers within the two sex QTLs with the transcriptome data of *S. paramamosain*. Markers from only one QTL (qSEX_32-b) were successfully annotated. Among the sex-related candidate genes, the 26S proteasome non-ATPase regulatory subunit 3 (PSMD3) was identified via marker1101. 26S proteasome is an essential egg coat lysin found in sperm that enables its penetration through the egg’s vitelline coat (Sutovsky, 2011). Low density lipoprotein receptor-related protein 2 (*LRP2*) gene was found via marker131703. LRP2 is postulated to be involved in the development of reproductive organ by regulating the uptake of androgen and estrogen bound to the sex-steroid binding globulin in reproductive tissues (Willnow et al., 2007).

### QTL for Growth-Related Traits

Quantitative trait loci mapping of growth-related traits has been conducted on various aquaculture species, including fish (Fu et al., 2016; Peng et al., 2016; Pang et al., 2017; Sun et al., 2017), shrimps (Andriantahina et al., 2013; Baranski et al., 2014), crabs (Cui et al., 2015; Hui et al., 2017; Lv et al., 2017) and bivalve mollusks (Wang et al., 2016; Nie et al., 2017). The high-density linkage maps constructed in the current study serve as a powerful tool for accurate QTL mapping, allowing a complete identification of the QTL locations and markers’ sequences, of which both are essential in the genetic improvement of selected traits in aquaculture (Andriantahina et al., 2013). This study is the first reported attempt of QTL mapping of growth-related traits in *S. paramamosain*. The measured growth traits of *S. paramamosain* were significantly correlated between one another and the QTL regions of almost all growth traits (90%), including some with high economic values such as CL, CW, and BW, were located on LG46. The distribution of almost all growth traits on one LG reflects the tight linkage of these traits whereas QTLs of these traits located on a small interval of 25.18 to 33.74 cM indicates that these traits may be regulated by the same genes occupying the same/nearby genetic positions (Andriantahina et al., 2013; Lv et al., 2017). This strongly indicates that LG46 serves as a major chromosome involved in growth regulation of *S. paramamosain*. The QTL clustering of almost all growth traits also explains for the positive correlation among the measurements of various growth traits. Future decoding of the full genome of *S. paramamosain* will allow the discovery of potential candidate genes influencing the QTLs of growth-related traits, especially on LG46, found in this study. Among the markers found in growth-related traits’ QTLs, only marker2388 on LG46 was successfully annotated to two genes – multidrug resistance-associated protein 4 (*MRP4*); cystic fibrosis transmembrane conductance regulator (*CFTR*). Interestingly, MRP4 is known to be involved in the regulation of prostaglandins across cell membranes (Reid et al., 2003). Prostaglandins is proven to affect molting of crustaceans, where higher prostaglandins resulted in shorter molt duration cycle in *Penaeus esculentus* (Koskela et al., 1992). This might suggest that MRP4 may have an indirect regulation on growth of mud crab by modulating the expression of prostaglandins, and thus molting.

### Association Analysis Between SNP Markers and Growth-Related Traits

The high number (67 out of 95) of SNP markers significantly associated with growth-related traits as indicated by GLM analysis reflects the results obtained via QTL analysis. The non-association of some SNP markers with their respective traits is expected, as the calculated PVE values for all markers only ranged between 5.8 and 11.95%. Two markers (i.e., 3846 and 7391) were significantly associated with the highest number of growth-related traits (16 out of 20) while seven markers were associated with more than two growth-related traits (Supplementary Table S10). Meanwhile, DLP4 trait was significantly linked to 38 markers. Such phenomenon where one marker was associated with several traits and several markers simultaneously associated with one trait indicates that one SNP marker might be involved in the regulation of several growth-related traits and several SNP markers are also potentially responsible in controlling the same trait in *S. paramamosain*. Similar observation was also reported in previous association studies using transcriptome-derived microsatellite markers with the growth performance in *S. paramamosain* (Ma et al., 2014) and in other aquaculture species, such as Asian seabass *Lates calcarifer* (Xu et al., 2006), large yellow croaker *Larimichthys crocea*(Xiao et al., 2016) and Pacific oyster *Crassostrea gigas* (Wang and Li, 2017). The polymorphisms and their potential regulatory effects on growth-related traits observed in this study highlight the involvement of potential genes in growth regulation of *S. paramamosain*. Thus, further study on the identification of these candidate genes based on the current QTL analysis data is recommended. Additionally, the replicability and correlation of the four markers associated with economically important growth traits across families, populations and generations should be investigated as genes are known to segregate and/or recombine over generations (Tizaoui and Kchouk, 2012). Based on our results, individuals with genotype hk serve as better candidates for future breeding programs based on their higher values of economically important growth traits and markers corresponded to each targeted trait could be used for the selection of *S. paramamosain* with higher growth performance.

### Applications of High-Density Linkage Maps and Growth-Related Traits QTLs in Genomics, Genetics, and Breeding

The constructed high-density linkage map of *S. paramamosain*, with a total length of 5,996.66 cM and an average marker interval of 0.81 cM, serves as a solid foundation for future genome sequencing, sequence assembly and marker-assisted selection of economically important traits. The putative ZW/ZZ sex determination system of *S. paramamosain* uncovered in this study contributes significantly toward the understanding of sex determination mechanism in decapod crustaceans and facilitates future establishment of mono-female culture population. Future research on QTLs of growth-related traits, especially on the QTL regions of CL, CW, and BW found in this study is expected to improve the breeding and aquaculture of *S. paramamosain*. In addition to being useful in promoting genetic breeding and stock enhancement, and to prevent inbreeding in the aquaculture sector, the linkage map constructed in the present study and the available SNP markers are also beneficial to population studies of wild mud crabs, including parentage assignment and population structure analysis (Smith et al., 2007; Panetto et al., 2017). Further, due to the limited number of genetic markers available for other *Scylla* species, the large number of SNP markers described in this study could potentially be amplified in other closely related species.

## Author Contributions

HM conceived and designed the research. HM, XS, and KW performed the research. HM, KW, XS, and SF analyzed the data. HM contributed reagents and materials. KW wrote the manuscript. SL, YZ, HZ, WL, and MI provided substantial comments and revised the manuscript. All authors read and approved the final version of the manuscript.

## Conflict of Interest Statement

The authors declare that the research was conducted in the absence of any commercial or financial relationships that could be construed as a potential conflict of interest.

## Footnotes

1 ftp://ftp.ncbi.nlm.nih.gov/blast/executables/blast+/LATEST/
