# Supplementary material for: High-Density Genetic Linkage Maps Provide Novel Insights Into ZW/ZZ Sex Determination System and Growth Performance in Mud Crab (Scylla paramamosain)
Source: Front Genet. 2019 Apr 5;10:298. doi: 10.3389/fgene.2019.00298 (PMC6459939; doi:10.3389/fgene.2019.00298)
Supplement: Supplementary file 1 [file Table_1.DOC]

**High-density genetic linkage maps provide novel insights into ZW/ZZ sex determination system and growth performance in mud crab (*Scylla paramamosain*)**

Khor Waiho^1,2,3^,**^†^**, Xi Shi^1,2^,**^†^**, Hanafiah Fazhan^1,2^, Shengkang Li^1,2^, Yueling Zhang^1,2^, Huaiping Zheng^1,2^, Wenhua Liu^1,2^, Shaobin Fang^1,2^, Mhd Ikhwanuddin^2,4^, Hongyu Ma^1,2,3,*^

**Supplementary Information includes:**

Supplementary Figure S1-S2

**Supplementary Figures**

**
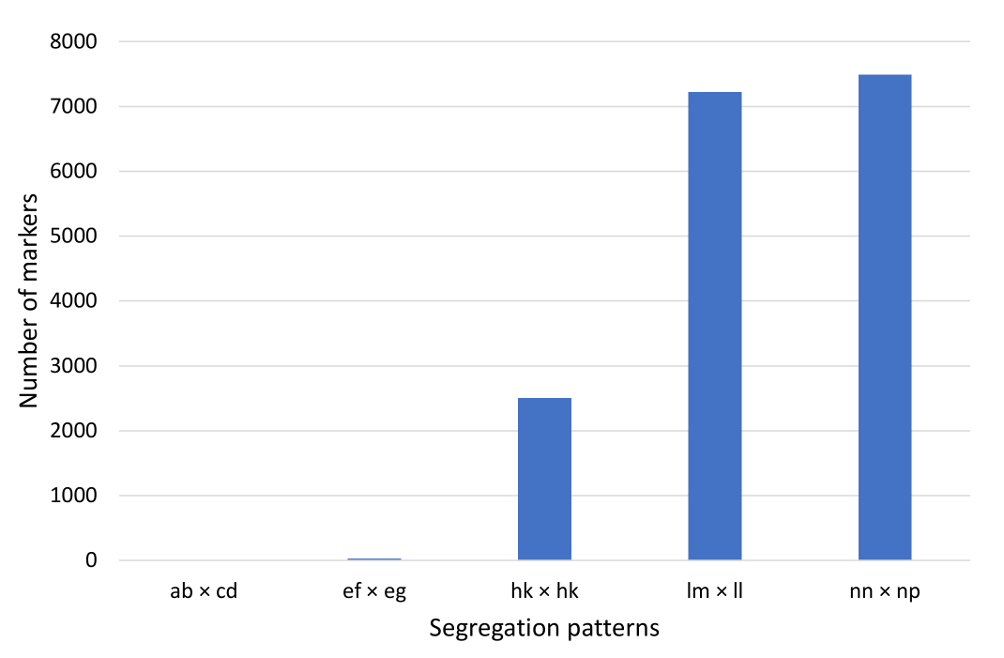
**

**FIGURE S1.** The number of markers corresponding to each segregation pattern.


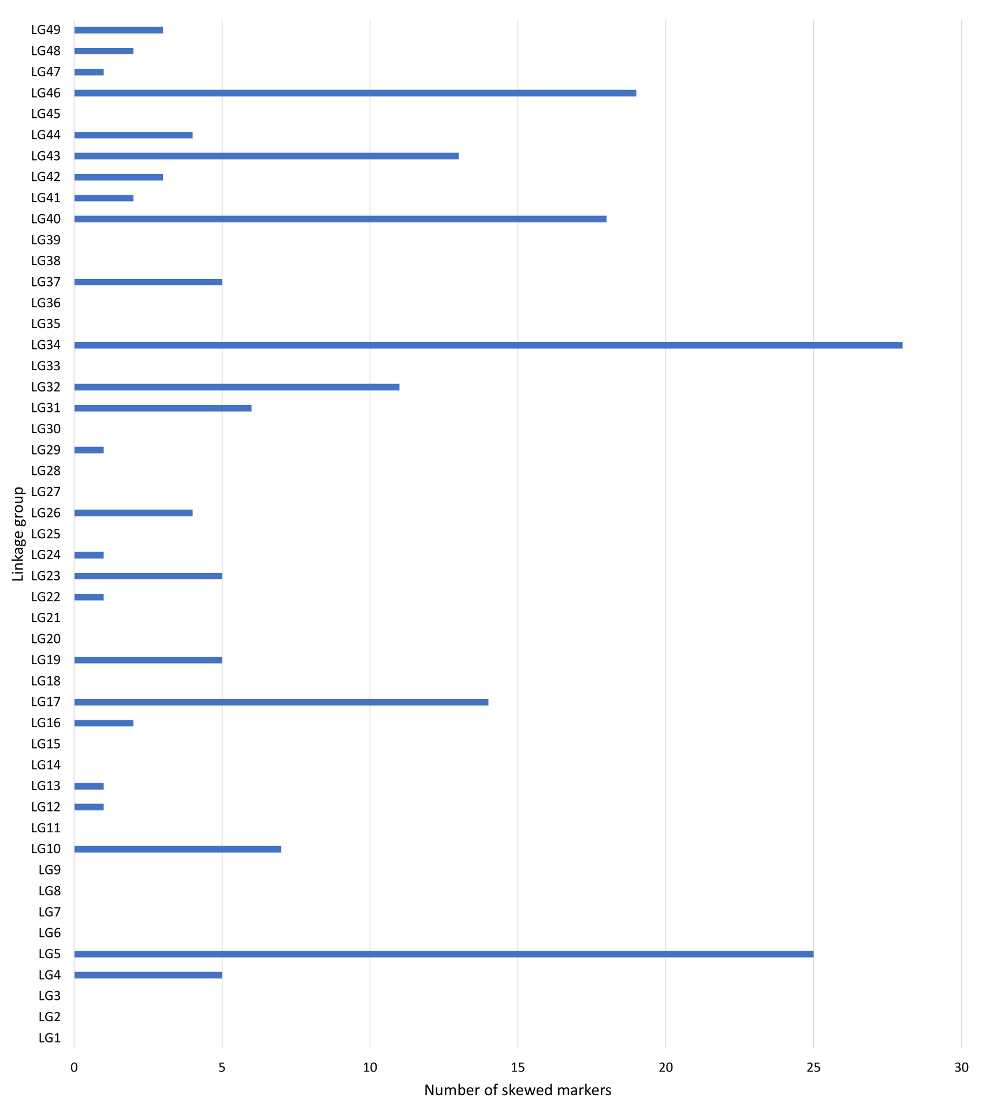


**FIGURE S2.** Significant segregation distortion (Chi square test; *p* < 0.05) of 187 markers in *Scylla paramamosain* linkage groups.
